# The Ability to Enhance the Solubility of Its Fusion Partners Is an Intrinsic Property of Maltose-Binding Protein but Their Folding Is Either Spontaneous or Chaperone-Mediated

**DOI:** 10.1371/journal.pone.0049589

**Published:** 2012-11-16

**Authors:** Sreejith Raran-Kurussi, David S. Waugh

**Affiliations:** Protein Engineering Section, Macromolecular Crystallography Laboratory, Center for Cancer Research, Frederick National Laboratory for Cancer Research, Frederick, Maryland, United States of America; University of Queensland, Australia

## Abstract

*Escherichia coli* maltose binding protein (MBP) is commonly used to promote the solubility of its fusion partners. To investigate the mechanism of solubility enhancement by MBP, we compared the properties of MBP fusion proteins refolded *in vitro* with those of the corresponding fusion proteins purified under native conditions. We fused five aggregation-prone passenger proteins to 3 different N-terminal tags: His_6_-MBP, His_6_-GST and His_6_. After purifying the 15 fusion proteins under denaturing conditions and refolding them by rapid dilution, we recovered far more of the soluble MBP fusion proteins than their GST- or His-tagged counterparts. Hence, we can reproduce the solubilizing activity of MBP in a simple *in vitro* system, indicating that no additional factors are required to mediate this effect. We assayed both the soluble fusion proteins and their TEV protease digestion products (i.e., with the N-terminal tag removed) for biological activity. Little or no activity was detected for some fusion proteins whereas others were quite active. When the MBP fusions proteins were purified from *E. coli* under native conditions they were all substantially active. These results indicate that the ability of MBP to promote the solubility of its fusion partners *in vitro* sometimes, but not always, results in their proper folding. We show that the folding of some passenger proteins is mediated by endogenous chaperones *in vivo*. Hence, MBP serves as a passive participant in the folding process; passenger proteins either fold spontaneously or with the assistance of chaperones.

## Introduction

The ability of certain highly soluble proteins to enhance the solubility of their fusion partners is often exploited for the production of recombinant proteins [1]. *Escherichia coli* maltose-binding protein (MBP) falls into this category and has been used extensively to circumvent inclusion body formation, particularly in *E. coli* where the poor solubility of recombinant proteins is a serious bottleneck [2], [3], [4]. However, the mechanism of fusion-mediated solubility enhancement remains poorly understood.

A variety of mechanisms, which are not necessarily mutually exclusive, have been proposed to explain how some but not all highly soluble proteins are able to function as solubility enhancers in the context of a fusion protein. One possibility is that solubility enhancers exert their effects by acting as “electrostatic shields”, reducing the probability of aggregation via electrostatic repulsion between highly charged soluble polypeptide extensions. While some solubility-enhancing fusion partners may function in this manner [5], this seems unlikely in the case of MBP because no correlation was observed between the net charges of MBPs from different microorganisms (all of which share a very similar fold) and their efficacy as solubility enhancers [6]. Another possible mechanism envisions the formation of soluble aggregates in which incompletely folded, hydrophobic passenger proteins occupy the center of a micelle-like sphere with hydrophilic domains shielding them from solvent. Indeed, there is good evidence for the formation of soluble, high molecular weight aggregates of human papilloma virus E6 fused to MBP [7]. How such seemingly “dead end” aggregates could evolve into properly folded fusion proteins remains unclear. Solubility enhancers have also been proposed to serve as “entropic anchors” by restricting the motion of a slow folding passenger protein and enabling it to fold in a more entropically favorable environment by reducing the number of possible conformations that can be sampled [8]. If this theory is correct, then any soluble (and folded) fusion partner would be expected to exert a similar entropic effect on the folding of the attached protein and promote its solubility, which is not the case. Neither the micelle nor the entropic-anchor model can readily account for the observation that only a subset of highly soluble proteins, such as MBP, are effective solubilizing agents. Yet another theory is that solubility-enhancing fusion partners act as “chaperone magnets” and solubility results from interactions with endogenous chaperones [9]. Finally, it has been proposed that solubility enhancers may have an innate, passive chaperone-like quality that manifests itself as iterative cycles of transient intramolecular binding to passenger proteins in a manner that prevents their self-association and aggregation [4], [10], [11], [12].

In an effort to illuminate the mechanism by which MBP, a universally acknowledged solubility-enhancing tag [13], [14], [15], [16], [17], promotes the solubility of its fusion partners, we have conducted refolding experiments with MBP fusion proteins *in vitro*. Additionally, we have examined how passenger proteins fold when fused to MBP, both *in vitro* and *in vivo*. Our results indicate that MBP has an intrinsic ability to solubilize its fusion partners that does not depend on any exogenous factors. Further, we present evidence that there are at least two pathways to the native state: passenger proteins either fold spontaneously or they are assisted by endogenous chaperones *in vivo*.

## Materials and Methods

### Construction of Expression Vectors

Various protein expression vectors were constructed by Gateway cloning (Life Technologies Inc., Carlsbad, CA), using the destination vectors pDEST-527, pDEST-565 (Protein Expression Laboratory, SAIC-Frederick, Frederick, MD, USA) and pDEST-HisMBP [18]. The standard LR reaction was employed throughout as per the manufacturer’s protocol. A two-step PCR procedure was used to construct Gateway entry clones of the passenger proteins. The open reading frames or entry clones encoding green fluorescent protein (GFP) [4], glyceraldehyde 3-phosphate dehydrogenase (G3PDH) [6], dihydrofolate reductase (DHFR) [6], dual specificity phosphatase 14 (DUSP14) [19], and tobacco etch virus (TEV) protease [20] were described previously. In each case, a pair of gene-specific primers was utilized in a PCR reaction with the appropriate plasmid template and then the PCR amplicon from this reaction was used as the template for a second round of PCR with the forward primer PE-277 (5′-GGGG ACA AGT TTG TAC AAA AAA GCA GGC TCG GAG AAC CTG TAC TTC CAG-3′) and the gene-specific reverse primer (Table 1). The final PCR amplicons were recombined into pDONR221 (Life Technologies) to generate the entry clones, except for GFP and G3PDH, which were recombined into pDONR201 (Life Technologies) instead. All of the entry clones were subsequently recombined in LR reactions with the destination vectors mentioned above. The resulting protein expression vectors encoded either His_6_- (pDEST-527 in the LR reaction), His_6_-GST (pDEST-565 in the LR reaction), or His_6_-MBP (pDEST-HisMBP in the LR reaction) tags appended to the N-termini of the passenger proteins along with canonical TEV protease recognition sites (ENLYFQG) between the tags and the passengers (except for the vectors encoding TEV protease fusions, which contained the uncleavable recognition site ENLYFQP [21] instead). The pDEST-HisMBP derivative carrying an I329W mutation in MBP was constructed with a QuikChange Site-Directed Mutagenesis Kit (Agilent Technologies Inc., Santa Clara, CA). The nucleotide sequences of all vectors were confirmed experimentally. GroEL/S plasmids used in co-expression and interaction studies were obtained from Jonathan Weissman’s laboratory [22].

**Table 1:** Primer sequences.

Passenger protein	Sequence (5' – 3')
G3PDH Forw.	GAGAACCTGTACTTCCAGGGTATGGTGAAGGTCGGTGTGAACGGATTTG
G3PDH Rev.	GGGGACCACTTTGTACAAGAAAGCTGGGTTATTACTCCTTGGAGGCCATGTAGGCCATGAGG
GFP Forw.	GAGAACCTGTACTTCCAGGGTGCTAGCAAAGGAGAAGAACTCTTC
GFP Rev.	GGGGACCACTTTGTACAAGAAAGCTGGGTTATTATTTGTATAGTTCATCCATGCCA
DHFR Forw.	GAGAACCTGTACTTCCAGGGTATGGTTGGTTCGCTAAACTGCATCGTCGC
DHFR Rev.	GGGGACCACTTTGTACAAGAAAGCTGGGTTATTAATCATTCTTCTCATATACTTCAAATTTG
DUSP14 Forw.	GAGAACCTGTACTTCCAGGGTATTTCCGAGGGTGACATCGGTGGCATTGCTCAAATCACC
DUSP14 Rev.	GGGGACCACTTTGTACAAGAAAGCTGGGTTATTAGTGTCGGGACTCCTTCTCATAGAC
TEV protease Forw.	GAGAACCTGTACTTCCAGCCGGAAAGCTTGTTTAAGGGGCCGCGTG
TEV protease Rev.	GGGGACCACTTTGTACAAGAAAGCTGGGTTATTAGCGACGGCGACGACGATTCATG

### Protein Expression, SDS-PAGE and Western Blot Analysis

Measurements of protein expression and solubility were performed essentially as described [4]. *E. coli* BL21-CodonPlus (DE3)-RIL cells (Agilent Technologies) were used for all expression experiments unless otherwise specified. *In vivo* expression studies involving His_6_-MBP-GFP and GroEL/S were performed in DH5α cells as described previously [22] with slight modifications. In brief, the His_6_-MBP-GFP expression vector and pJDW66 (or its derivative encoding the GFP-optimized GroEL/S variant 3–1, pJDW67) were co-transformed into *E. coli.* A single fresh colony was inoculated into LB broth with appropriate antibiotic(s) and grown at 37°C. His_6_-MBP-GFP expression was induced in log phase cultures (OD_600_ = 0.2–0.4) by the addition of IPTG to 1 mM. The cells were harvested after 3 h, resuspended in 50 mM Tris-HCl (pH 7.6), 1 mM EDTA and disrupted by sonication. The cells expressing His_6_-MBP-GFP and GroE_wt_ (or GroE_3–1_) were normalized by final cell OD_600_, illuminated under blue light (fluorescence) or visible light and photographed. Samples of the total and soluble intracellular protein for SDS-PAGE were extracted from these normalized cell suspensions and the gels were stained with Coomassie brilliant blue R-350.

Wild-type or otherwise isogenic single gene knockout mutants of *E. coli* K-12 (*ΔdnaK*, *ΔdnaJ*, *Δtig*) [23], [24] were used for expression studies involving His_6_-MBP-G3PDH and His_6_-MBP-DHFR, which were performed as described above.

Immunoblotting was carried out using standard procedures with anti-Histag (Abcam, Cambridge, MA) or anti-GroEL (Sigma-Aldrich, St. Louis, MO) primary antibodies and alkaline phosphatase (AP)-conjugated secondary antibodies (KPL, Gaithersburg, MD).

### Purification of Proteins

Proteins were purified from 1–2 L cultures using Talon columns (Clontech Laboratories Inc., Mountain View, CA). Bacterial cell pellets were resuspended in 50 mM sodium phosphate (pH 7.0), 6 M guanidine hydrochloride (Gdn-HCl), 300 mM NaCl, 15 mM imidazole (buffer A), stirred for 1 h at room temperature, and then sonicated. After centrifugation at 30,000 *g* for 20 min, the supernatant was collected and loaded onto two tandem 5 ml Talon columns equilibrated in buffer A. The columns were washed to baseline with 50 mM sodium phosphate (pH 7.0), 8 M Urea, 300 mM NaCl, 15 mM imidazole (buffer B) and then eluted with linear gradient from 15 to 250 mM imidazole in buffer B. The fractions containing the protein of interest were collected and concentrated using Amicon stirred cells (EMD Millipore, Billerica, MA). The purification of MBP fusion proteins under native conditions was performed as described elsewhere [25]. The GroEL and GroES used for *in vitro* refolding studies were purified as described [26], [27].

### *In vitro* Refolding

Purified proteins were refolded by the rapid dilution (1∶50 v/v) method with three additions of the same volume over a 32 h period into refolding buffer. The refolding conditions were different depending on the passenger protein. We used conditions previously reported to support efficient refolding of G3PDH [28], and DHFR [29]. Renaturation of GFP was carried out in a refolding buffer containing 0.1 M sodium phosphate pH 7.4, 2 mM EDTA, 5 mM DTT and 0.5 M L-arginine hydrochloride. The standard reaction buffer for TEV protease (50 mM Tris-HCl, pH 8.0, 0.5 mM EDTA, 1 mM DTT) with 0.5 M L-arginine hydrochloride was used for refolding TEV protease. For DUSP14, a simple Tris-buffered saline solution that worked well for the related enzyme DUSP6 [30] was used. Additions were performed drop-wise with stirring, and then the solution, which remained clear, was incubated at 4°C for 10–12 h. This material was ultrafiltered using an Amicon YM10 membrane (Millipore) and the retentate (10–15 mL) was centrifuged (30,000 *g*/4°C/20 min) to remove the precipitate, if any. The soluble proteins were buffer exchanged into PBS (pH 7.4) by extensive dialysis.

The concentration and total yield of the refolded fusion proteins were determined spectrophotometrically on the basis of their absorbance at 280 nm (A_280 nm_) and calculated extinction coefficients. However, because some preparations contained a significant amount of truncated polypeptides, fusion protein concentrations were also assessed by comparing the Commassie Blue staining intensity of serial dilutions with known quantities of BSA after SDS-PAGE (data not shown).

The His_6_-MBP-DHFR and His_6_-MBP-G3PDH fusion proteins were also refolded in the presence of purified GroEL and GroES. The refolding buffer contained a 2-fold molar excess of GroES (1.2 µM) relative to GroEL (0.6 µM). The final concentration of the enzymes (G3PDH and DHFR) was kept at 0.3 µM. Refolding was initiated by the addition of ATP to 5 mM along with 10 mM MgCl_2_. The solution was mixed, and after 15 min at room temperature, enzyme activity was analyzed.

### Enzyme Assays and GFP Fluorescence Quantitation

The enzymatic assays for the passenger proteins were conducted essentially as reported previously for G3PDH [31], DHFR [32], and DUSP14 [33]. Briefly, for G3PDH, the assay mixture contained 5.6 mM 3-phosphoglycerate, 1 mM ATP, 300 µM NADH, 5 mM MgSO_4_, 1 mM EDTA, 1 mM DTT, and 50 µg phosphoglycerate kinase/ml. The change in absorbance at 340 nm was followed for 2 min after addition of the enzyme. The DHFR enzyme activity was analyzed by the decrease in the NADPH concentration detected spectrophotometrically at 340 nm upon addition of the enzyme. The reaction mix contained 50 mM Tris-HCl (pH 7.4), 5 mM MgCl_2_, 3.3 mM KCl, 10 mM DTT, 0.1 mM dihydrofolate, and 0.1 mM NADPH and was monitored for 5 min. DUSP14 activity was measured by using para-nitrophenylphosphate (pNPP) as the substrate in a reaction mix containing 50 mM Bis-Tris (pH 6.8,) 1 mM EDTA, 1 mM DTT, and 10% DMSO. The enzyme was added to the reaction mix and incubated at 37°C for 10 min. The reaction was terminated by the addition of 3 N NaOH and the developed color was read at 405 nm.

The enzymatic activity of TEV protease was analyzed by digesting an MBP-NusG fusion protein substrate [34] in 50 mM Tris-HCl, pH 8.0, 0.5 mM EDTA, and 1 mM DTT. The reaction was incubated at room temperature for 10 min. A 1∶10 molar ratio of enzyme:substrate was used. The reaction was stopped by the addition of 2X SDS-PAGE sample buffer and the digestion products were analyzed by SDS-PAGE. The gel was stained with Coomassie Blue and the results were quantified with a CCD camera and Alpha Imager software (Alpha Innotech, San Leandro, CA).

Fluorescence spectra for GFP were measured with a spectrofluorometer FluoroMax-2 (Jobin Yvon HORIBA-SPEX, Edison, NJ). The concentration of GFP was 0.8 µM in all the fluorescence measurements. All the measurements were made at 25°C using appropriate blanks for baseline correction of fluorescence intensity. The emission maximum at 508 nm was used for calculating relative units.

In all of the above enzymatic assays, either commercially available pure enzymes from Sigma-Aldrich or ProSpec (East Brunswick, NJ) or crystallization-grade pure proteins that were produced in our laboratory were used as reference standards. Relative values were obtained by normalization against the reference standards. All chemicals used were of analytical grade.

## Results

### Design of Fusion Proteins

**Figure 1:**
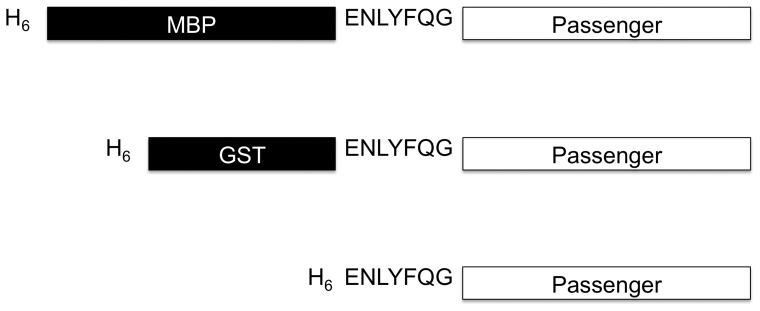
Design of fusion proteins. Schematic representation of fusion proteins with three different N-terminal tags: H_6_, H_6_-GST, and H_6_-MBP (not to scale). In the tagged forms of TEV protease, the canonical TEV protease recognition site (ENLYFQG) was replaced by an uncleavable recognition site ENLYFQP [21] to prevent autodigestion of the fusion proteins.

To investigate the mechanism of solubility enhancement by MBP, we conducted a series of refolding experiments with MBP fusion proteins. The five passenger proteins selected for these experiments (G3PDH, GFP, DHFR, TEV protease, and DUSP14) represent diverse origins, functions, and physiochemical properties. Importantly, all of them are insoluble when expressed in an unfused form or as GST fusion proteins in *E. coli* [2,4,6, and unpublished results]. Moreover, they all have enzymatic activities (or fluorescence emission in the case of GFP) that can be used to monitor their folding. The MBP used in these experiments had a polyhistidine tag appended to its N-terminus so that the fusion proteins could be purified under denaturing conditions. The N-terminal His-tag does not interfere with the ability of MBP to promote the solubility of its fusion partners [18]. As controls, the same passenger proteins were also fused to His_6_-GST, a poor solubility enhancer, and His_6_ alone to approximate the unfused state. The G3PDH, GFP, DHFR and DUSP14 fusion proteins included a recognition site for TEV protease (ENLYFQG) adjacent to the N-termini of the passenger proteins (Figure 1). The three tagged forms of TEV protease instead included an uncleavable recognition site (ENLYFQP) [21] to prevent autodigestion.

### Refolding of Fusion Proteins

**Figure 2:**
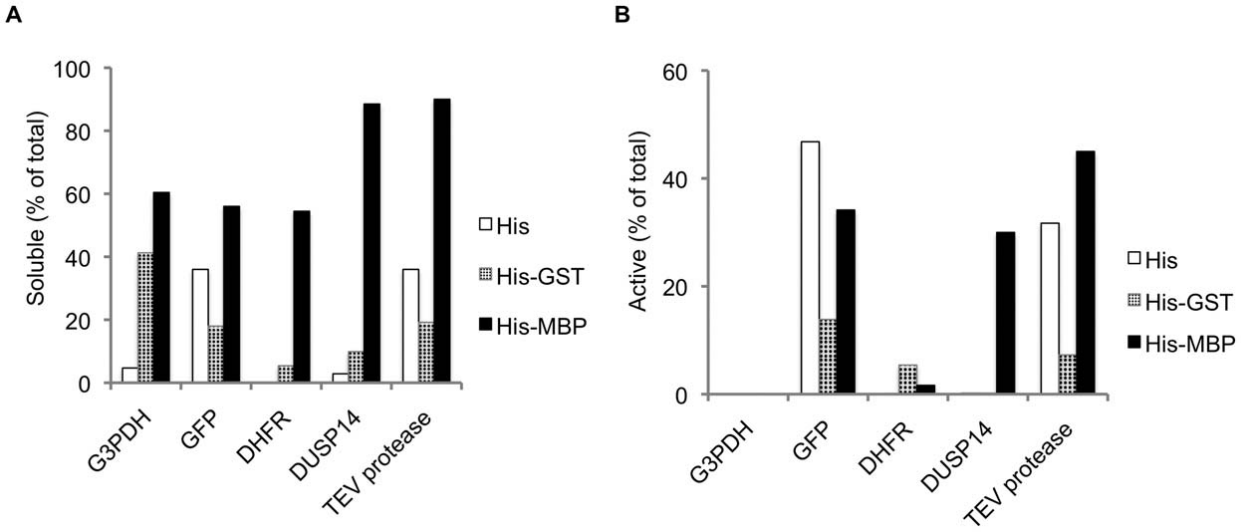
Yield and activity of soluble fusion proteins after refolding. The yield of soluble fusion protein (A) and active passenger protein (B) was calculated and expressed as a percentage of the total amount of protein added to the refolding reactions.

The His_6_, His_6_-GST and His_6_-MBP fusion proteins were refolded by rapid dilution, after which aggregates were removed by ultrafiltration and centrifugation. Remarkably, all of the His_6_-MBP fusions yielded substantially more soluble protein after refolding than did the corresponding His_6_-GST- or His_6_-tagged fusions (Figure 2A), mirroring the same trend that was observed when these fusion proteins were expressed in *E. coli* [2], [4], [6]. Because prior experiments suggested that the open (apo) conformation of MBP mediates solubility enhancement [25], refolding of MBP fusion proteins was also performed in the presence of 30 mM maltose. However, this did not affect the amount of soluble protein that was recovered (data not shown).

To assess the status of the passenger proteins after refolding, we performed enzyme assays or fluorescence measurements in the case of GFP on the intact (uncleaved) fusion proteins. Although the yield of soluble His_6_- and His_6_-GST fusion proteins was much lower than the yield of the His_6_-MBP fusion proteins, in all cases there was still enough material to assay. We calculated the fraction of the soluble protein that was active and report it as a percentage of the total protein added to the refolding reactions (Figure 2B). The results revealed that roughly equivalent amounts of GFP and TEV protease were obtained when these two passenger proteins were fused to His_6_-MBP or to His_6_ alone, indicating that MBP did not influence the folding of these proteins but only increased their solubility. Interestingly, the His_6_-GST-TEV and His_6_-GST-GFP fusion proteins were significantly less active, suggesting that the His_6_-GST tag actually impedes the folding of TEV protease and GFP. On the other hand, the folding of DUSP14 was greatly stimulated as a consequence of being fused to MBP, suggesting a more active role for MBP in the folding of this passenger. The activity of the other two passenger proteins, DHFR and G3PDH, was negligible irrespective of their N-terminal fusion partner.

When the soluble G3PDH, GFP, DHFR and DUSP14 fusion proteins were cleaved by TEV protease, the majority of the passenger proteins precipitated (no effort was made to cleave the TEV protease fusion proteins because they lacked functional protease recognition sites). However, in all cases, the MBP and GST domains remained soluble and were folded because they could be quantitatively adsorbed onto amylose and glutathione resin, respectively (data not shown). Taken together, these results demonstrate that the ability of MBP to promote the solubility of its fusion partners *in vitro* does not always result in their proper folding, as has also been observed *in vivo* [7].

### Folding of Fusion Proteins in vivo

When the His_6_-MBP fusion proteins were purified under native conditions, we found that all of them were highly active, some even more so than the standards obtained from commercial sources (Table 2). The difference was greatest for the passenger proteins DHFR and G3PDH. Remarkably, the His_6_-MBP-DHFR and His_6_-MBP-G3PDH fusion proteins exhibited levels of enzymatic activity that were consistent with 100% folding whereas the same fusion proteins had negligible activity after refolding. This led us to conclude that additional factor(s) must participate in the folding of these passenger proteins in *E. coli.*

**Table 2:** Specific activity of refolded vs. natively purified fusion proteins.

Passenger protein	Relative specific activity or relative emission max_508 nm_ of MBP fusions	
	*In vitro* refolded	Natively purified (*In vivo*)
G3PDH	0.00	1.77
DHFR	0.03	1.37
DUSP14	0.34	0.97
TEV protease	0.50	0.50
GFP	0.73 (Relative emission max_508 nm_)	1.26 (Relative emission max_508 nm_)

### Involvement of Chaperones in the Folding of Fusion Proteins

**Figure 3:**
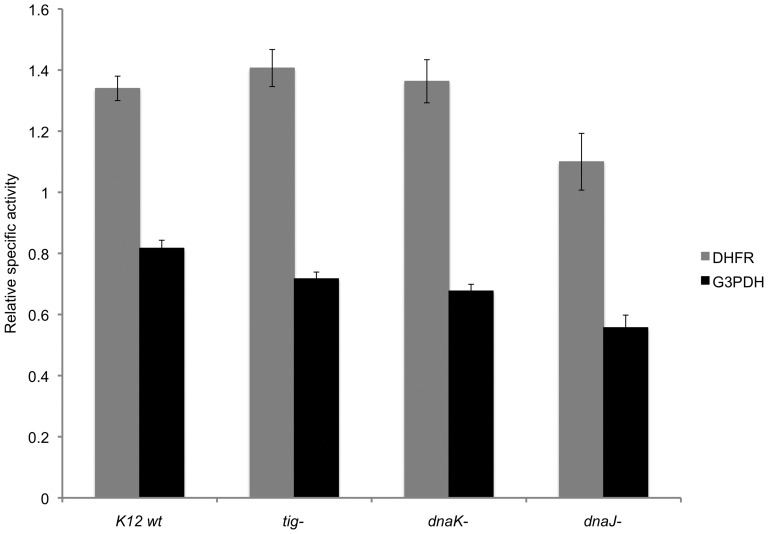
The effect of *dnaJ*, *dnaK* and *tig* gene deletions on the enzymatic activity of MBP-DHFR and MBP-G3PDH fusion proteins purified under native conditions. The data with error bars are expressed as mean ± standard error of the mean (n = 3). The relative values were obtained by normalization with a standard protein in each case.

Reasoning that molecular chaperones might be the endogenous factors required for the folding of some fusion proteins in *E. coli,* we next investigated the potential role of DnaK, DnaJ and trigger factor in this process by purifying the His_6_-MBP-DHFR and His_6_-MBP-G3PDH fusion proteins under native conditions from “wild-type” *E. coli* K12 and otherwise isogenic strains containing deletions of the corresponding genes. The results revealed that the absence of these chaperones resulted in only a modest reduction in the yield of properly folded DHFR and G3PDH; not nearly enough to account for the difference between the activities observed *in vitro* and *in vivo* (Figure 3).

Intriguingly, we observed that natively purified His_6_-MBP-G3PDH and His_6_-MBP-DHFR were always contaminated with GroEL (Figure S1). However, very little GroEL was found to be associated with natively purified His_6_-MBP itself (Figure S1, lane 3), suggesting that the chaperonin was binding to the passenger proteins. Yet co-purification of GroEL with fusion proteins is not uncommon and is generally interpreted as being indicative of protein misfolding [35]. Therefore, this observation does not prove that GroEL actively assists with the folding of the fusion proteins. In fact, because MBP is a relatively large fusion partner (42 kDa), it is doubtful that most MBP fusion proteins could fit inside the “Anfinsen cage” of the chaperonin, which has been estimated to be capable of housing proteins up to 70 kDa in principle, with the actual size exclusion limit being somewhat less [36].

**Figure 4:**
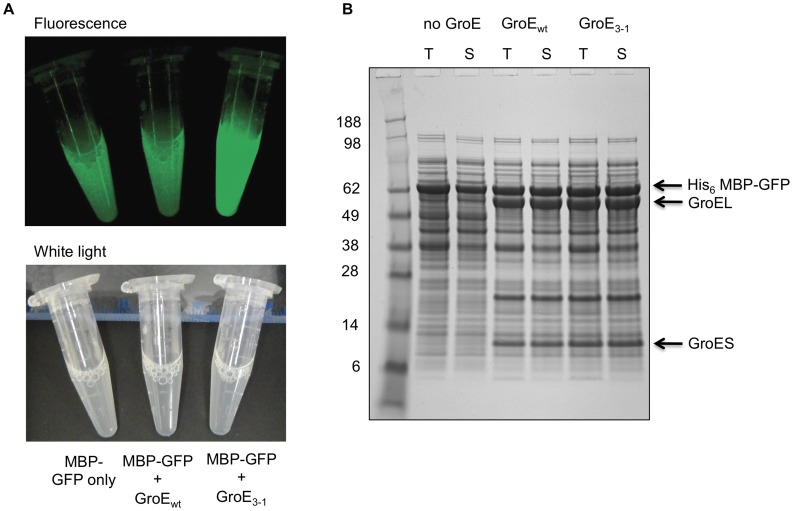
Interaction of MBP fusion proteins with GroEL/S. (**A**) Lysed cells co-expressing H_6_-MBP-GFP and either wild-type GroE or the GroE_3–1_ variant are shown under blue or white light illumination. Cells co-expressing GroE_3–1_ fluoresce more intensely than cells co-expressing wild-type GroE as a result of enhanced GFP folding. Cells expressing only the MBP-GFP fusion protein are shown on the left. (**B**) SDS-PAGE analysis of total and soluble proteins from the cells in (A). T, total intracellular protein; S, soluble intracellular protein.

To ascertain whether MBP fusion proteins are capable of interacting productively with GroEL/S *in vivo*, we took advantage of a GroEL/S mutant (GroE_3–1_) generated by directed evolution that is far more effective at stimulating the folding of GFP than is the wild-type chaperonin [22]. When GroE_3–1_ was co-expressed with the His_6_-MBP-GFP fusion protein (∼70 kDa), the cells were significantly more fluorescent than they were when the wild-type chaperonin was co-expressed with the fusion protein or when only the fusion protein was overexpressed (Figure 4A). The increased fluorescence in the cells with GroE_3–1_ was a result of enhanced GFP folding because co-expression of GroE_3–1_ or wild-type GroE did not alter the amount of His_6_-MBP-GFP fusion protein that was produced (Figure 4B). Similar results were obtained when the even larger solubility enhancing tag NusA (∼55 kDa) was joined to GFP to create an 82 kDa fusion protein (Figure S2).

### *In vitro* Refolding of MBP Fusions with GroEL/S

**Figure 5:**
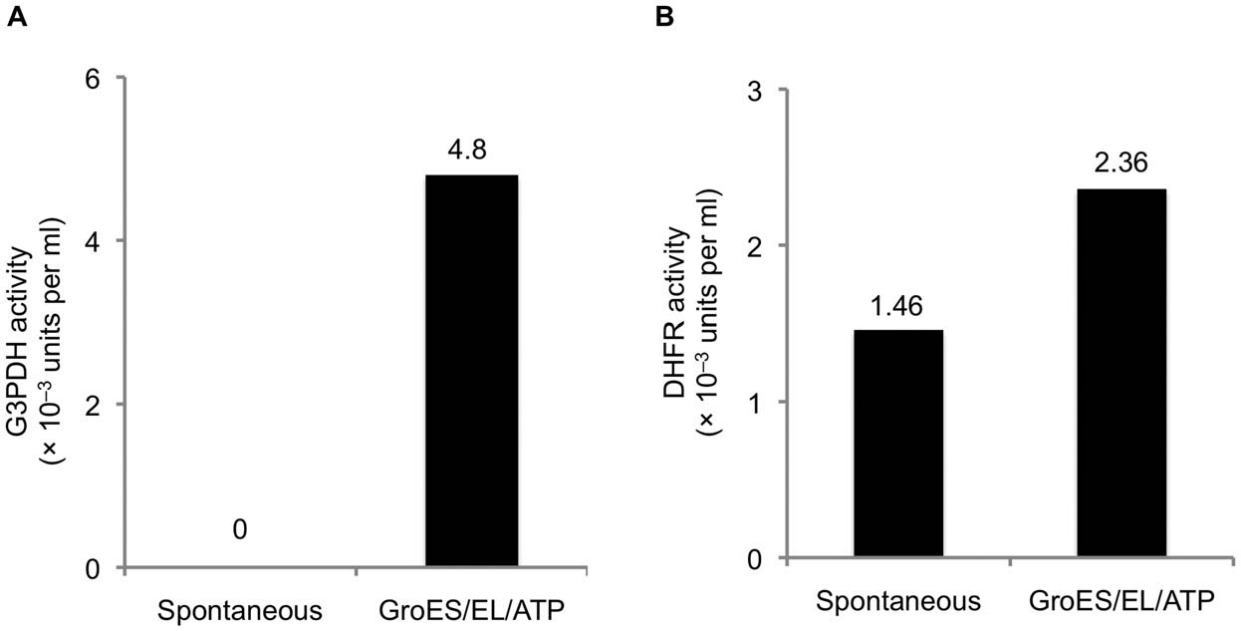
The addition of GroEL and GroES increases the yield of properly folded passenger proteins *in vitro*. (**A**) G3PDH activity. (**B**) DHFR activity.

Seeking to confirm that the GroEL/S chaperonin is involved in the folding of DHFR and G3PDH when these proteins are expressed as His_6_-MBP fusions in *E. coli*, we next performed *in vitro* refolding experiments in the presence of purified GroEL and ATP/Mg^2+^. The addition of GroEL alone did not improve the recovery of active passenger proteins in these cases (data not shown). However, the addition of GroES along with GroEL and ATP/Mg^2+^clearly stimulated the folding of both DHFR and G3PDH (Figure 5). These results are consistent with the hypothesis that GroEL/S plays an active role in the folding of the G3PDH and DHFR fusion proteins in *E. coli*.

### Interaction of Other Fusion Proteins with GroEL/S in E. coli

**Figure 6:**
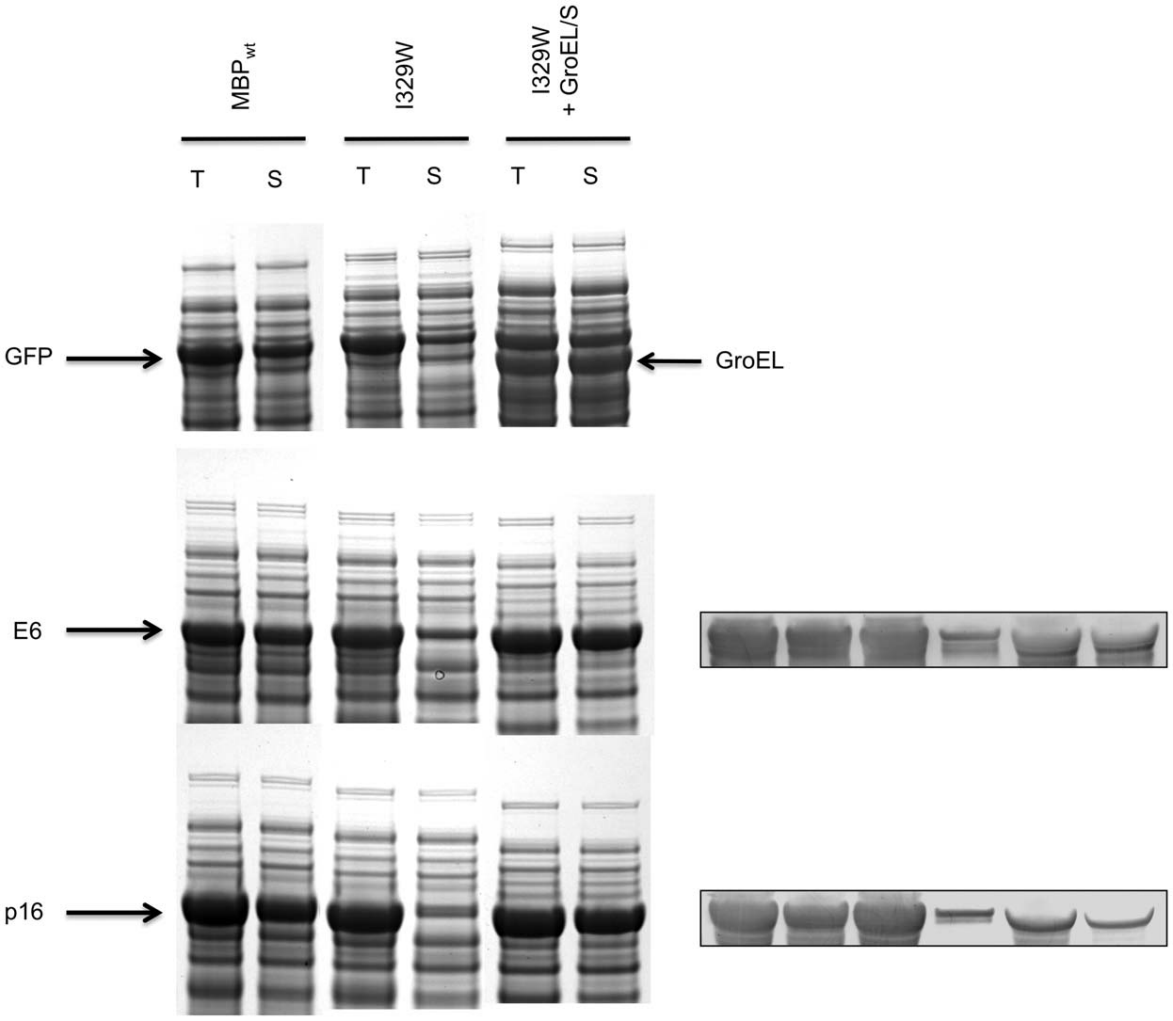
Overproduction of GroEL/S rescues the solubility defects of some MBP fusion proteins. Expression and solubility of wild type MBP (MBP_wt_) and mutant MBP (I329W) fusion proteins are shown in the figure. The co-expression of GroEL/S along with mutant MBP fusions rescues the solubility (right most pair of lanes). The passenger proteins were GFP (top), E6 (middle) and p16 (bottom). A Western blot using anti-His_6_ tag antibody is shown to the right since the fusion proteins and GroEL co-migrates in the case of E6 and p16 (MBP fusion proteins carry a His_6_ tag at the N-terminus); loading is similar to the respective gels on the left.

It was previously shown that a single amino acid substitution in MBP (I329W) dramatically decreases the solubility of several fusion proteins in *E. coli* but has no impact on the solubility of MBP in its unfused state [25]. The phenotype of this mutation was attributed to its effect on the equilibrium between the “open” and “closed” conformations of MBP, the latter being inhibitory to solubility enhancement. Intriguingly, we have found that the solubility defects of these fusion proteins can be rescued in whole or in part by co-expression of the GroEL/S chaperonin (Figure 6). Although the explanation for this effect remains to be elucidated, it constitutes further circumstantial evidence for an interaction between GroEL/S and MBP fusion proteins in *E. coli*. Moreover, the involvement of additional passenger proteins (e.g., human papilloma virus E6 and the tumor suppressor p16^INK4a^) suggests that the interaction of MBP fusion proteins with GroEL/S *in vivo* is not restricted to DHFR and G3PDH and may be a relatively common phenomenon.

## Discussion

### The Mechanism of Solubility Enhancement by MBP

The present study clearly demonstrates that the extraordinary ability of MBP to promote the solubility of its fusion partners is innate: no extraneous factors are necessary to elicit this effect *in vitro*. This finding agrees with an earlier observation that the recovery of soluble procapthepsin D and pepsinogen after refolding could be enhanced by fusing them to MBP [37], and confirms the generality of this result. Exactly why MBP is such an effective solubility enhancer (in contrast to many other highly soluble proteins) remains uncertain, but the fact that it can perform this feat *in vitro* appears to rule out the “chaperone magnet” model. Consistent with an earlier report [38], the experiments described here support a role for the chaperonin GroEL/S in the folding of some passenger proteins but not in solubility enhancement by MBP. Rather, our results indicate that chaperones and/or chaperonins seem to come into play after a passenger protein has been rendered soluble by MBP.

Kapust and Waugh suggested that MBP functions as a kind of passive chaperone in the context of a fusion protein [4]. Iterative cycles of binding and release by MBP of partially folded passenger proteins eventually results in their spontaneous folding while avoiding the kinetically competing self-aggregation pathway. The hydrophobic ligand-binding pocket in MBP, which is not present in other highly soluble proteins that do not function as solubility enhancers (e.g., GST), was proposed to be the locus of polypeptide binding. The phenotypes of some mutations in MBP were observed to be consistent with this model [25]. However, one might then expect that the occupation of this pocket by maltose, which results in the transition from an “open” to a “closed” complex [39], would impede solubility enhancement by MBP. Yet, at odds with this prediction, we found that the inclusion of as much as 30 mM maltose in refolding experiments did not appreciably reduce the recovery of soluble MBP fusion proteins (MBP has a *K_D_* of 1200 nM for maltose [40]). This does not necessarily rule out the intramolecular chaperone model, however, because the proposed interaction site may lie elsewhere on the surface of MBP [8].

### Two Pathways for the Folding of Passenger Proteins

We have shown that there are at least two pathways to the native state for passenger proteins that have been rendered soluble by fusing them to MBP. Some proteins such as TEV protease and GFP can fold spontaneously if their propensity to form insoluble aggregates is blocked by fusing them to MBP. Other passenger proteins, exemplified by G3PDH and DHFR, depend on endogenous GroES/L to fold correctly after being solubilized by MBP. In both cases, MBP serves as a kind of “holdase” to maintain the passenger proteins in an aggregation-resistant form that either permits spontaneous folding to occur or affords access to molecular chaperones.

Among the passenger proteins examined in the present study, DUSP14 represents a unique case because its folding pathway differs in at least one respect from those described above. Although DUSP14 folds *in vitro* in the absence of chaperones, the yield of active enzyme on a mole-per-mole basis is far greater as an MBP fusion protein than as a His_6_-GST or His_6_-tagged protein (Figure 2B). This contrasts with GFP and TEV protease, which exhibit similar mole-per-mole refolding yields with the various tags and therefore appear to undergo spontaneous rather than MBP-assisted folding. The unusual behavior of DUSP14 suggests the existence of yet another possible pathway for passenger protein folding that is more directly dependent on MBP.

Co-expression experiments conducted with the MBP-GFP and NusA-GFP fusion proteins in the presence of the GroE_3–1_ variant unequivocally demonstrate that proteins larger than the theoretical volume of the cavity formed by a GroEL heptamer can engage in productive folding interactions with the chaperonin. Moreover, a cell-wide survey of GroEL/S clients identified several proteins larger than 60 kDa [41], [42]. It is now generally accepted that these large substrates/clients utilize a so-called “trans” mechanism in which they occupy one of the two cavities in the back-to-back dimer of GroEL heptamers while the other empty cavity binds the co-chaperonin GroES and ATP, enabling conformational changes to be propagated from one cavity to the other [43], [44]. One needs to bear in mind that even though we have emphasized the interaction of passenger proteins with GroEL/S, it is also possible that the chaperonin interacts with MBP as well [45]. We have found GroEL co-purifying with MBP on an affinity (IMAC) column (Figure S1A, lane 3) and the solubility rescuing effect observed upon co-expression of the GroES/L chaperonin with mutant MBP (I329W) fusion proteins (Figure 6) is also suggestive of an interaction with MBP.

**Figure 7:**
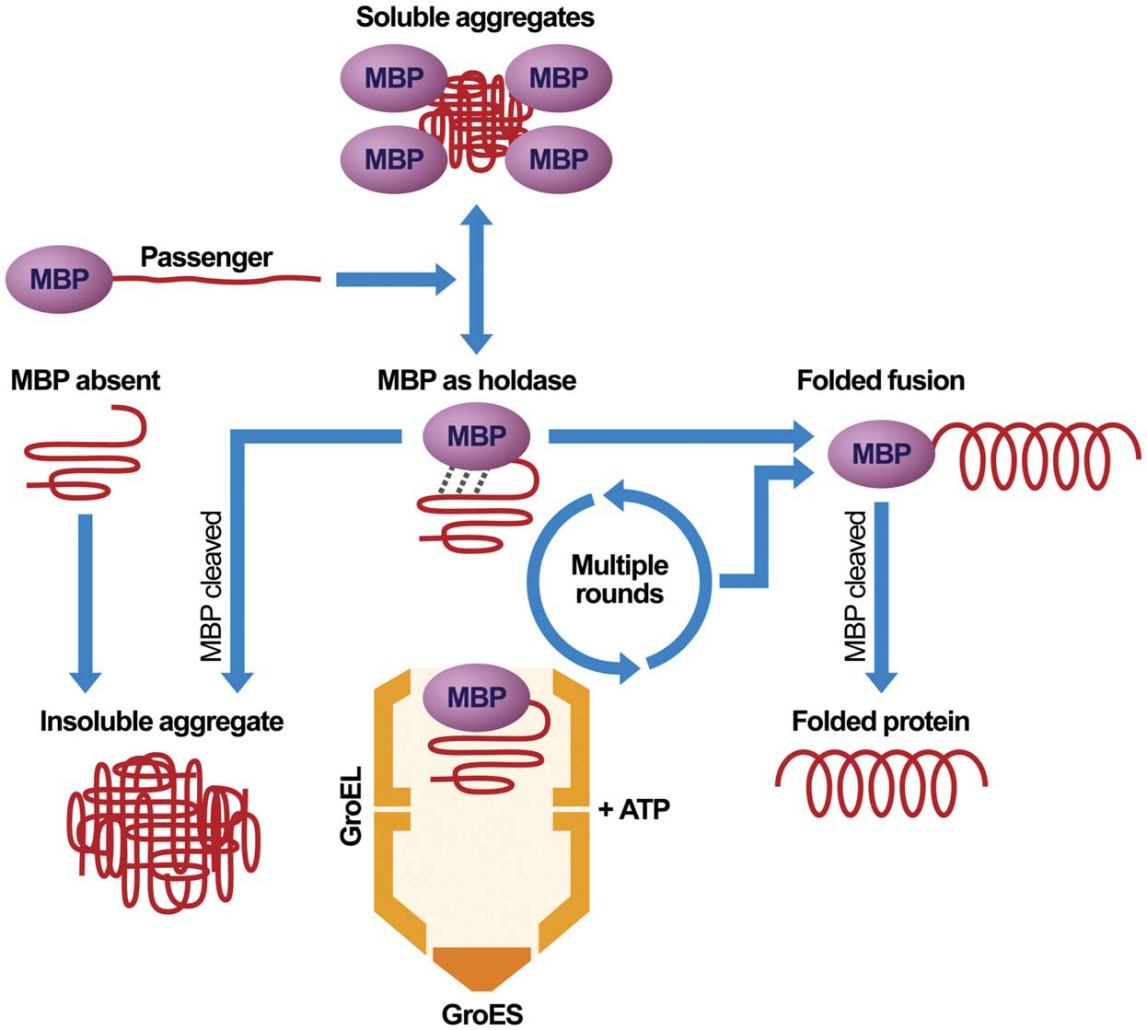
A model illustrating the roles that MBP plays in the production of recombinant proteins (see text for discussion).

Based on the experiments reported here, along with the results of previous work [4], [7], [8], [25], [37], [38], 46, we propose the model for solubility enhancement and folding that is depicted in Figure 7. A protein that normally accumulates in the form of insoluble aggregates when expressed in an unfused form in *E. coli* (MBP absent) is prevented from doing so when fused to MBP (MBP as holdase). Exactly how MBP promotes the solubility of its fusion partners is unknown but this may involve a transient physical interaction between a folded MBP moiety and an incompletely folded passenger protein. Our refolding experiments confirm the existence of such partially folded intermediates. The incompletely folded passenger protein may engage in multiple rounds of binding to and release from MBP. Some passenger proteins reach their native conformation by spontaneous folding after one or more cycles, while in other cases MBP facilitates the interaction between an incompletely folded passenger protein and one or more endogenous chaperones. In both cases, MBP serves primarily as a “holdase”, keeping the incompletely folded passenger protein from forming insoluble aggregates until either spontaneous or chaperone-mediated folding can occur. A third class of passenger proteins is unable to fold via either of these pathways and exists perpetually in an incompletely folded state, either as an intramolecular or intermolecular (i.e., micelle-like) aggregate. These passenger proteins typically precipitate after they are cleaved from MBP by a site-specific protease [46].

The utilization of MBP as a “holdase” during the production of recombinant proteins may be of considerable practical value in some cases. For instance, it may be fruitful to co-express GroEL/S along with MBP fusion proteins in cases when the yield of active recombinant protein is poor in spite of MBP tagging. Even though co-expression of GroEL/S with His_6_-MBP-G3PDH and His_6_-MBP-DHFR did not lead to any appreciable enhancement of enzymatic activity (Figure S3), indicating that endogenous chaperone levels were sufficient to fold all of the passenger protein in these instances, the yield of other passenger proteins might be improved by this approach. It would also be of interest to examine the effect of co-expressing various types of eukaryotic chaperones on the folding of MBP fusion proteins in *E. coli*. Conversely, because solubility enhancement is an intrinsic property of MBP, the production of MBP fusion proteins in eukaryotic expression systems might yield favorable results. Recently, MBP has also been used to maintain proteins that contain disulfide-bonds in a soluble state in the *E. coli* cytoplasm so that they could be acted upon by appropriate redox enzymes that were co-expressed in the same cellular compartment [47]. It seems likely that additional ways of exploiting the “holdase” activity of MBP for the production of recombinant proteins will be forthcoming.

## Supporting Information

Figure S1 **Copurification of GroEL with natively purified MBP fusions on an affinity (IMAC) column. (A)** Western blot using anti-GroEL antibody. Lane 1, His_6_-MBP-G3PDH; lane 2, His_6_-MBP-DHFR; lane 3, His_6_-MBP; lane 4, purified GroEL. **(B)** SDS-PAGE analysis of the above samples (loading same as above).
(TIF)

Figure S2 **Interaction of NusA fusion proteins with GroEL/S. (A)** Lysed cells co-expressing His_6_-NusA-GFP and either wild-type GroE or the GroE_3–1_ variant are shown under blue or white light illumination. Cells co-expressing GroE_3–1_ fluoresce more intensely than cells co-expressing wild-type GroE as a result of enhanced GFP folding. Cells expressing only the His_6_-NusA-GFP fusion protein are shown on the left. **(B)** SDS-PAGE analysis of total and soluble proteins from the cells in (A). T, total intracellular protein; S, soluble intracellular protein.
(TIF)

Figure S3 **Enzymatic activity from cells co-expressing GroEL/S and His_6_-MBP-fusions. (A)** G3PDH activity. **(B)** DHFR activity. The data with error bars are expressed as mean ± standard error of the mean (n = 3). Extracts from “wild-type” *E. coli* K-12 were prepared by sonication from equal amounts of cells expressing GroEL and GroES (pGroEL/S) or His_6_-MBP-fusions (G3PDH or DHFR) alone, or fusion proteins with GroEL/S (pGroEL/S+His_6_-MBP-G3PDH or His_6_-MBP-DHFR). The extracts were centrifuged at 14000 *g* for 10 min, and the soluble fraction was assayed for enzymatic activity.
(TIF)
