# Supplementary figures and images for: The Ability to Enhance the Solubility of Its Fusion Partners Is an Intrinsic Property of Maltose-Binding Protein but Their Folding Is Either Spontaneous or Chaperone-Mediated
Source: PLoS One. 2012 Nov 16;7(11):e49589. doi: 10.1371/journal.pone.0049589 (PMC3500312; doi:10.1371/journal.pone.0049589)

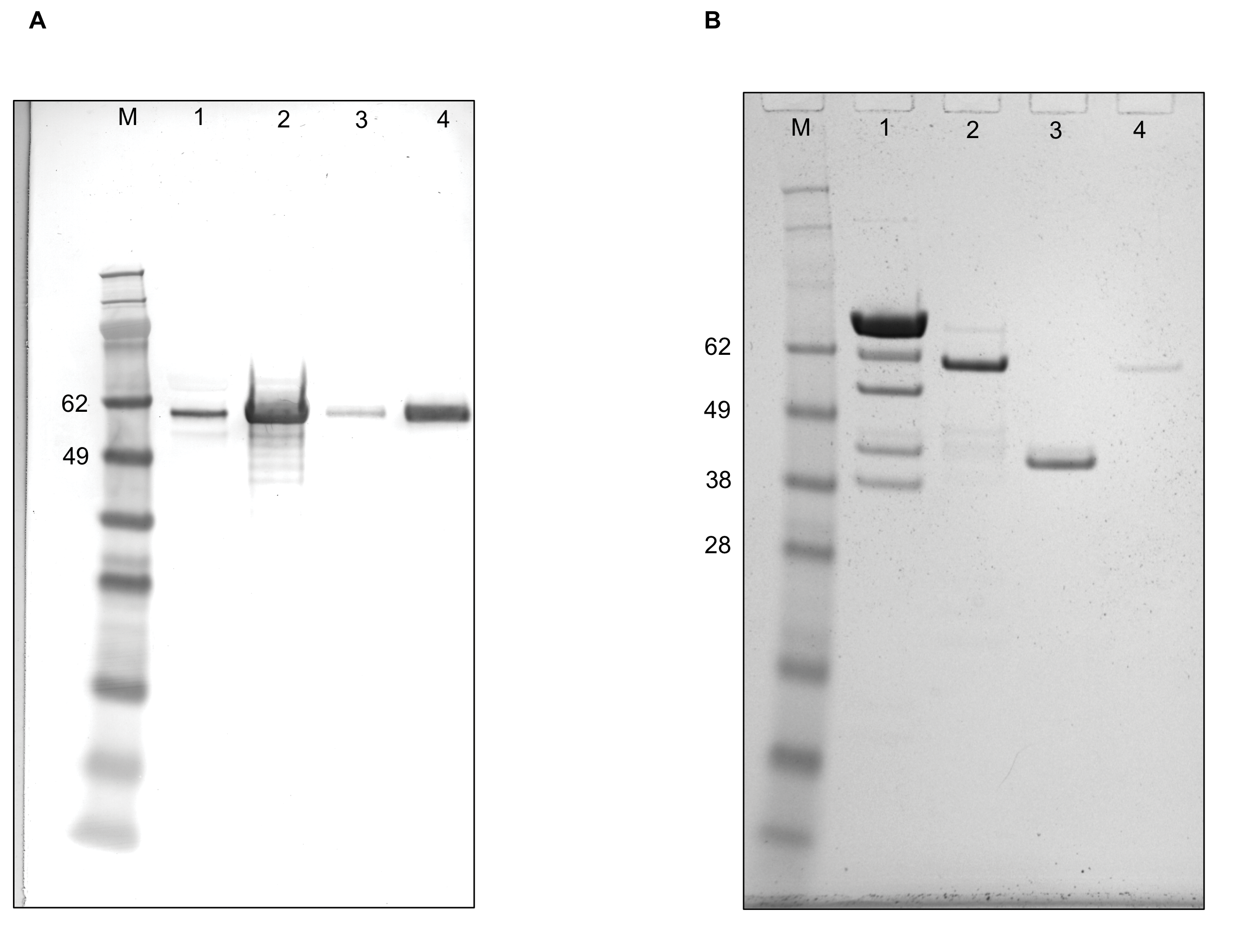

Supplement: Figure S1 — Copurification of GroEL with natively purified MBP fusions on an affinity (IMAC) column. (A) Western blot using anti-GroEL antibody. Lane 1, His6-MBP-G3PDH; lane 2, His6-MBP-DHFR; lane 3, His6-MBP; lane 4, purified GroEL. (B) SDS-PAGE analysis of the above samples (loading same as above). (TIF) [file pone.0049589.s001.tif]

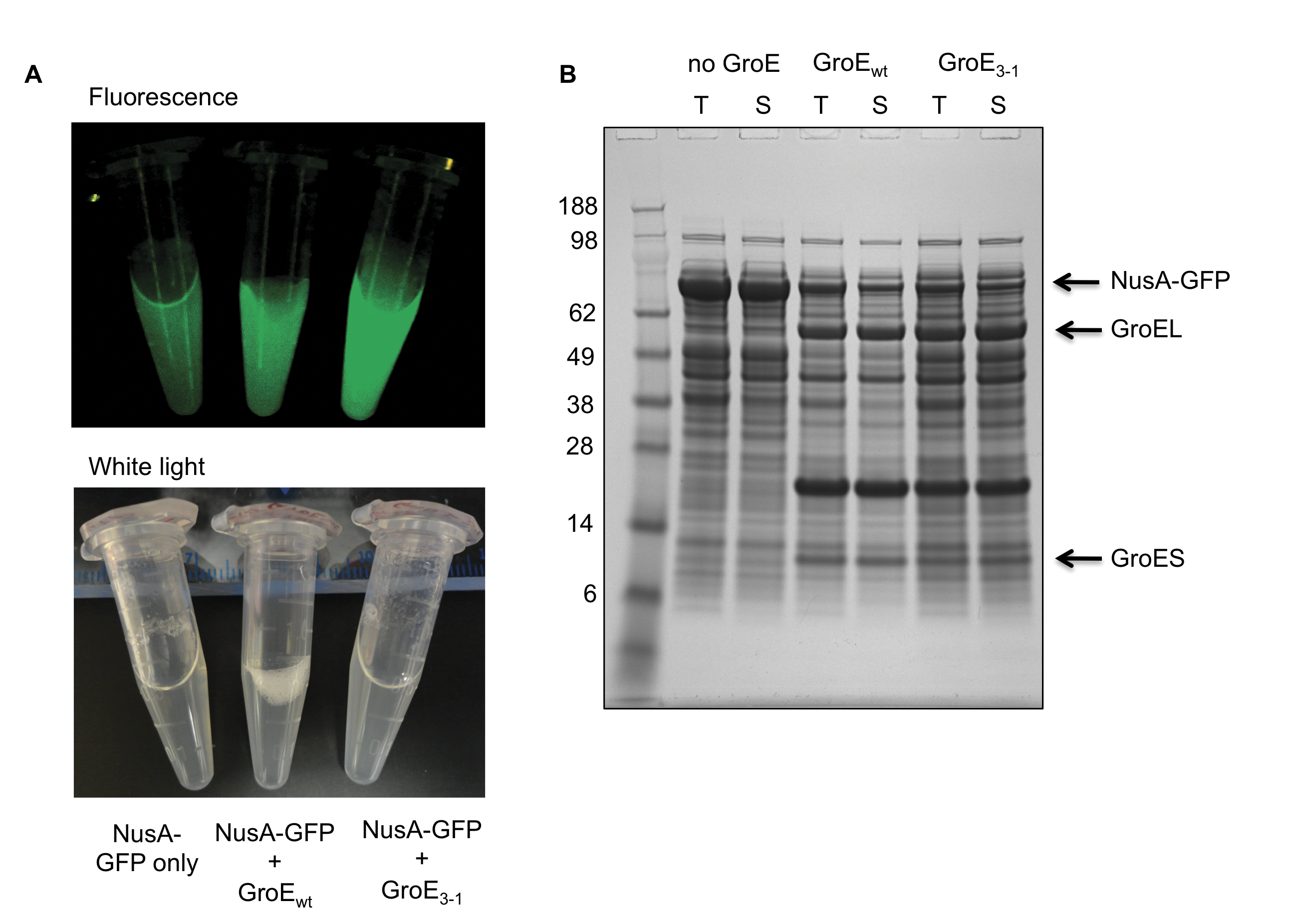

Supplement: Figure S2 — Interaction of NusA fusion proteins with GroEL/S. (A) Lysed cells co-expressing His6-NusA-GFP and either wild-type GroE or the GroE3–1 variant are shown under blue or white light illumination. Cells co-expressing GroE3–1 fluoresce more intensely than cells co-expressing wild-type GroE as a result of enhanced GFP folding. Cells expressing only the His6-NusA-GFP fusion protein are shown on the left. (B) SDS-PAGE analysis of total and soluble proteins from the cells in (A). T, total intracellular protein; S, soluble intracellular protein. (TIF) [file pone.0049589.s002.tif]

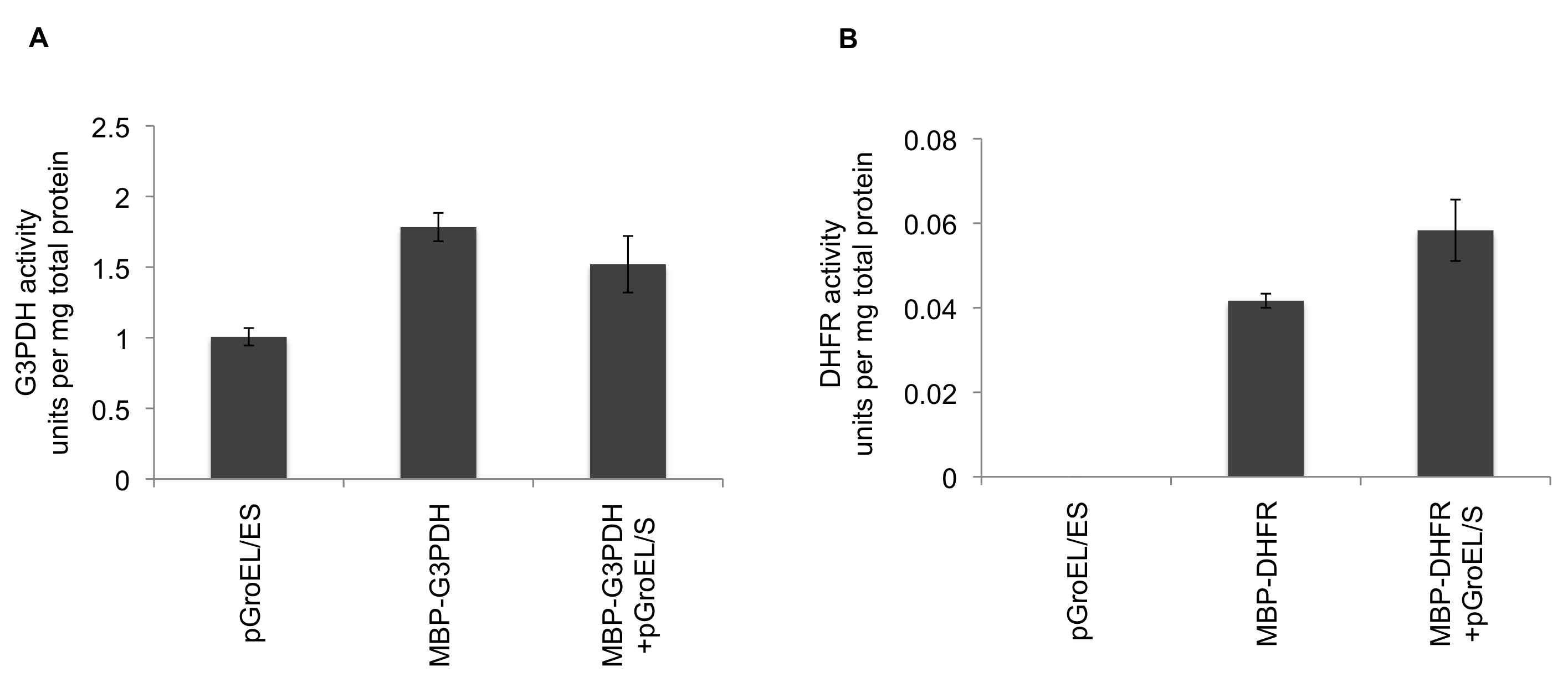

Supplement: Figure S3 — Enzymatic activity from cells co-expressing GroEL/S and His6-MBP-fusions. (A) G3PDH activity. (B) DHFR activity. The data with error bars are expressed as mean ± standard error of the mean (n = 3). Extracts from “wild-type” E. coli K-12 were prepared by sonication from equal amounts of cells expressing GroEL and GroES (pGroEL/S) or His6-MBP-fusions (G3PDH or DHFR) alone, or fusion proteins with GroEL/S (pGroEL/S+His6-MBP-G3PDH or His6-MBP-DHFR). The extracts were centrifuged at 14000 g for 10 min, and the soluble fraction was assayed for enzymatic activity. (TIF) [file pone.0049589.s003.tif]
